# Supplementary material for: Transcriptome Analysis Reveals the Response Mechanism of Frl-Mediated Resistance to Fusarium oxysporum f. sp. radicis-lycopersici (FORL) Infection in Tomato
Source: Int J Mol Sci. 2022 Jun 25;23(13):7078. doi: 10.3390/ijms23137078 (PMC9267026; doi:10.3390/ijms23137078)
Supplement: Supplementary file 1 [file ijms-23-07078-s001.zip › ijms-1767590-supplementary.pdf]

软件名字	开发者姓名	城市，国家
RSEM	Bo Li ， Colin N Dewey	Madison,USA
DESeq2	Michael Love, Wolfgang Huber, Simon Anders	Heidelberg, Germany
HISAT2	Daehwan Kim, Ben Langmead, Steven L Salzberg	Maryland, USA
Bowtie 2	Ben Langmead and Steven L Salzberg	Maryland, USA
SOAPnuke	Yuxin Chen	Shenzhen, China
mapman	Oliver Thimm, Mark Stitt	Golm, Germany
